# sRAGE inhibits myocardial ischemia/reperfusion injuries via regulating Treg cells

**DOI:** 10.1186/s13578-025-01482-y

**Published:** 2025-10-22

**Authors:** Jian Liu, Jie Zhang, Jing Chang, Lu Chen, Hongxia Wang, Yu Liu, Mingcong Huang, Xiangjun Zeng, Caixia Guo

**Affiliations:** 1 Cardiovascular Center, Beijing Tongren Hospital, Capital Medical University, No.1 Dongjiaomin Lane, Dongcheng District, Beijing, 100730 People’s Republic of China; 2 Department of Physiology, Beijing Youan Hospital, Capital Medical University, No. 8 You An Men Wai Xi Tou Tiao, Fengtai District, Beijing, 100069 People’s Republic of China; 3 Department of Physiology and Pathophysiology, Capital Medical University, No. 10 You An Men Wai Xi Tou Tiao, Fengtai District, Beijing, 100069 People’s Republic of China

**Keywords:** I/R, Myocardium, Differentiation, sRAGE, Tregs

## Abstract

**Background:**

Regulatory T cells (Tregs) have been documented to accumulate in damaged myocardial tissue, where they play a pivotal role in attenuating excessive inflammatory responses during myocardial ischemia/reperfusion (I/R) injury. Concurrently, soluble receptor for advanced glycation end-products (sRAGE) has been demonstrated to alleviate myocardial I/R injury by suppressing inflammation, suggesting a potential involvement of Tregs in the inhibitory effects of sRAGE on myocardial I/R injury.

**Methods:**

I/R surgery or glucose deprivation/reoxygenation was employed to explore myocardial injury and the related mechanisms by using cardiomyocyte-specific sRAGE knock-in mice or cultured cardiomyocytes. Potential molecular mechanisms were analyzed via western blotting, immunohistochemistry, and flow cytometric analysis.

**Results:**

The findings revealed that sRAGE overexpression significantly increased the numbers of Tregs. Depletion of Tregs abrogated the protective effects of sRAGE against I/R-induced cardiac dysfunction, myocardial fibrosis, and inflammatory response in cardiac-specific sRAGE transgenic mice. Mechanistically, sRAGE was found to enhance the expression of programmed cell death ligand 1 (PD-L1) and its upstream JAK2/STAT3 signaling axis, thereby facilitating CD4^+^ T cells differentiation into Tregs within myocardial tissue during I/R.

**Conclusions:**

The study demonstrated that sRAGE protected against myocardial I/R injury by modulating the differentiation of Tregs through upregulation of the JAK2/STAT3-PD-L1 signaling pathway.

**Supplementary Information:**

The online version contains supplementary material available at 10.1186/s13578-025-01482-y.

## Introduction

Myocardial ischemia/reperfusion (I/R) injury, a significant complication frequently encountered during percutaneous coronary intervention in the management of patients with acute myocardial infarction (AMI) [1, 2], is associated with a complex array of mechanisms, including mitochondrial dysfunction, excessive reactive oxygen species (ROS) production, and dysregulated inflammation [3]. Among these, the inflammatory response plays a pivotal role in determining the ultimate size of the myocardial infarct [4]. Therefore, inhibiting excessive and persistent inflammation after AMI would avoid subsequent left ventricular remodeling and heart failure [3, 4].

In tracing the mechanisms for inflammatory response, regulatory T cells (Tregs) are confirmed to suppress the inflammatory response after AMI [5], which would contribute to the maintenance of immune homeostasis and immune tolerance during myocardial I/R injury [6, 7]. Tregs, which can be recruited from circulation [6, 8], proliferated from resident Tregs [7], or differentiated from CD4 positive cells [9, 10], have been shown to mitigate myocardial injury by secreting cytokines and modulating the M1/M2 macrophage ratio during myocardial I/R injury [11]. Consequently, finding an appropriate way to increase Tregs in cardiac tissue would be wise to inhibit myocardial I/R injury.

Soluble receptor for advanced glycation end products (sRAGE), is the circulating isoform of the receptor for advanced glycation end products [12]. Previous studies have shown that sRAGE increases the number of Tregs in mice [12] and confers protection against myocardial I/R injury through multiple mechanisms, including attenuation of inflammation, reduction of apoptosis, and inhibition of myocardial fibrosis [13–15]. Additionally, sRAGE has been reported to promote the differentiation of CD4^+^ T cells into Tregs in cerebral I/R injury by inhibiting fatty acid synthesis [16]. However, the role of sRAGE in promoting CD4^+^ T cell differentiation into Tregs and its subsequent impact on myocardial I/R injury remains to be elucidated.

The differentiation of CD4^+^ T cells into Tregs is intricately linked to the programmed death-1 (PD-1)/programmed cell death ligand 1 (PD-L1) signaling axis [17]. PD-1 is expressed on immune cells [17], and programmed cell death ligand 1 PD-L1 is present on non-hematopoietic cells, such as vascular endothelial cells and cardiomyocytes [18]. The transcription of PD-L1 is regulated by Janus kinase 2 (JAK2)/signal transducers and activators of transcription (STAT) pathway [19–21]. Our prior research has demonstrated that sRAGE activates the JAK2/STAT pathway in cardiomyocytes during myocardial I/R injury [22]. Based on these findings, we hypothesize that sRAGE may augment Tregs numbers by modulating PD-L1 expression in cardiomyocytes via the JAK2/STAT pathway during myocardial I/R injury.

In the present study, we postulated that sRAGE mitigated myocardial I/R injury by promoting the differentiation of CD4^+^ T cells into Tregs within the myocardial tissue, a process mediated by the JAK2/STAT-PD-L1 signaling pathway.

## Materials and methods

### Generation of humanized sRAGE allele knock-in mice

Humanized sRAGE allele knock-in (sRAGE KI^fl/fl^) and Myh6-Cre mice (Cyagen-Bioscience Co., Ltd., China) were used, both maintained on a C57/B6J genetic background. The human RAGE transcript variant 6 (NM_001206940.2), commonly referred to as the sRAGE isoform, was selected for genomic insertion using the CRISPR/CAS9 system. Offspring were genotyped using PCR and sequencing analysis, with the primers used for genotype identification provided in Supplementary Table 1. Cardiomyocyte-specific sRAGE knock-in (sRAGE-CKI) mice were generated through crosses sRAGE KI^fl/fl^ mice with the Myh6-Cre mice. Male sRAGE-CKI mice (6–8 weeks old) were administered an intraperitoneal injection of tamoxifen (20 mg/kg/d) for 5 consecutive days before the I/R operation and maintained for 7 days to induce sRAGE expression [23]. All animal experiments were approved by the Animal Experimentation Ethics Committee of Capital Medical University and were conducted according to the Guide for the Care and Use of Laboratory Animals (National Institutes of Health).

### Myocardial I/R mice model

Myocardial I/R surgery was performed as previously described [14]. Briefly, mice were anesthetized via inhalation of 2% isoflurane and 100% oxygen at a flow rate of 2 L/min. Following skin incision and sequential tissue dissection, the thoracic cavity and heart were exposed. Myocardial ischemia was induced by transient ligation of the left anterior descending artery with a 6 − 0 silk suture for 30 min, followed by reperfusion achieved by releasing the slip knot. Mice were sacrificed by intraperitoneal injection of an overdose of sodium pentobarbital (100 mg/kg) followed by cervical dislocation. The harvested hearts were used for further study.

### Echocardiography

At the end of reperfusion, the mice were anesthetized by inhalation of 2% isoflurane and immobilized in a supine position. The mice underwent transthoracic echocardiography with a Vevo 2100 instrument (Visual Sonics, Toronto, Canada) to analyze cardiac structural and functional changes. Standard long-axis and short-axis M-mode images were recorded. Cardiac parameters, including left ventricular ejection fraction (LVEF), shortening fraction (FS), left ventricular cardiac output (CO), left ventricular end-diastolic volume (LVEDV), left ventricular end-systolic volume (LVESV) and heart rate (HR), were calculated using Vevo Lab 3.1.1 software.

### Analysis of myocardial infarct size

After reperfusion for 24 h, myocardial infarct size was assessed using 2,3,5-triphenyltetrazolium chloride (TTC) staining. TTC powder (Sigma Aldrich, St. Louis, MO, USA) was dissolved in phosphate-buffered saline (PBS) to prepare a 1% TTC solution. The heart was incubated in a 1% TTC solution at 37 °C for 5 min, subsequently fixed in 4% paraformaldehyde for 4 min, and then frozen at − 20 °C for 5 min. Cross-sections in the coronal plane were observed and photographed by stereomicroscope (Olympus SZX7, Tokyo, Japan). The white area in the heart represents infarcted cardiac tissue, while the red area represents non-infarcted tissue. Infarct area was quantified using Image J software and was expressed as a percentage of the total ventricular area.

### Flow cytometric analysis

After 36 h of reperfusion, the mice were sacrificed, with spleens, and hearts were harvested. Single-cell suspensions of spleen tissue were prepared by mechanical disruption with RBC lysis buffer. Cardiac tissue single-cell suspensions were obtained by mincing and enzymatic dissociation with 500U/mL using collagenase in HBSS solution at 37 °C for 1 h. The isolated cells were incubated with BV421-labeled anti-CD4 antibody (562891, BD Biosciences, San Diego, CA, USA), PE-Cy7-labeled anti-CD25 antibody (25025182, eBioscience, San Diego, CA, USA) and PE-labeled anti-Foxp3 antibody (12577382, eBioscience) for 1 h at room temperature. The stained cells were detected with the BD Verse flow cytometer (BD Bioscience, San Jose, USA), and the data were analyzed by FlowJo software (TreeStar, Oregon, USA).

### Immunohistochemical analysis

Hearts were excised and fixed in 10% neutral buffered formalin overnight. Tissues were then embedded in paraffin and sectioned into 4.0-µm slices using a sliding microtome. Immunohistochemical staining was performed according to the DAB assay kit (GK600710, Genetech, Shanghai, China). Sections were incubated with appropriate antibodies (Supplementary Table 2), and further processed using the DAB kit for colorimetric detection. To identify Tregs in the cardiac tissue, double immunostaining for CD4 and Foxp3 was performed utilizing the Alkaline Phosphatase (mouse IgG), VECTASTAIN ABC-AP kit (AK-5002, Vector Laboratories, Burlingame, CA, USA) and Vector Red (SK-5100, Vector Laboratories), according to the manufacturer’s instructions. Hematoxylin was used to stain the cell nuclei. Images of the cardiac tissues were captured via a digital slide scanner (Pannoramic SCAN, 3DHISTECH, Budapest, Hungary) and analyzed using Image J software.

### Isolation and treatment of neonatal rat cardiomyocytes

Neonatal rat cardiomyocytes were isolated from the heart of 1-day-old Sprague-Dawley rat. Briefly, hearts were immersed in Ca^2+^Mg^2+^-free HBSS solution containing 0.25% trypsin at 37 °C. The supernatant was transferred to an equal volume of Dulbecco’s modified eagle’s medium/nutrient mixture F-12 ham (DMEM-F12; D8437, Sigma-Aldrich, St Louis, MO, USA) supplemented with 15% fetal bovine serum to terminate the enzymatic digestion. The mixture was centrifuged at 1000 rpm for 10 min, after which the supernatant was discarded, and the cell pellet was resuspended in the fresh medium. After being incubated for 90 min to remove fibroblasts, the supernatant was collected and seeded into 6-well plates, where the cells were cultured at 37 °C in a 5% CO_2_ incubator for further experimentation. After being starved for 6 h, cardiomyocytes were infected with adenovirus carrying either sRAGE particles (Ad-sRAGE) or Ad-GFP as a control for a minimum of 24 h. Infected cells were subjected to oxygen-glucose deprivation (OGD) by treatment with “ischemic buffer” in a tri-gas incubator (37 °C, 1% O₂, 5% CO₂, 94% N₂) for 2 h, followed by reperfusion with DMEM-F12 and culture in a standard 5% CO₂ incubator for 24 h to complete the OGD/reoxygenation (OGD/R) protocol. The compositions of “ischemic buffer” have been described previously.

### Extraction of naive CD4^+^ T cells

Wild-type C57/BL6J mice were euthanized via intraperitoneal injection of tribromoethanol. Spleens were aseptically extracted and processed to obtain single-cell suspensions, from which CD4⁺ T cells were enriched using a lymphocyte separation solution (7211011, Dakewe, Shenzhen, China). Following cell quantification, a Biotin Antibody Cocktail and Anti Biotin Micro Beads were proportionally added (130-104-453, Miltenyi, Bergisch-Gladbach, German). The suspension was then subjected to magnetic bead sorting to isolate naive CD4^+^ T cells.

### Western blot analysis

Heart, spleen tissues and, cardiomyocytes were lysed in RIPA lysis buffer (R0010, Solarbio, Beijing, China) supplemented with protease inhibitors. Lysates were centrifuged at 4 °C for 15 min, and the supernatants were collected as the total protein sample. The same amount of proteins (50 µg) from each group was separated by SDS-PAGE (P1200, Solarbio), and transferred to PVDF membranes (PVDF; IPVH00010, Millipore, Billerica, MA, USA). Membranes were blocked with 5% skim milk for 1 h before incubation with primary antibodies (Supplementary Table 2) at 4℃ overnight. The following day, the membranes were incubated with HRP-conjugated secondary antibody goat anti-rabbit IgG for 1 h at room temperature. Protein bands were visualized on a chemiluminescence imaging system (FluorChem FC3, ProteinSimple, Santa Clara, CA, USA) with the chemiluminescent HRP substrate (WBKLS0500, Millipore, Billerica, MA, USA). Protein levels were normalized to Tubulin or GAPDH loading controls, respectively.

### Statistical analyses

Data were presented as mean ± SEM. The data were analyzed by ANOVA with the Least Significant Difference (LSD) test or the Games-Howell test for comparison involving more than two groups. Statistical significance was defined as *P* < 0.05. All analyses were conducted using SPSS v20.0 software (IBM SPSS, Chicago, IL, USA).

## Results

### sRAGE increased the number of Tregs in myocardial tissue during I/R

**Fig. 1:**
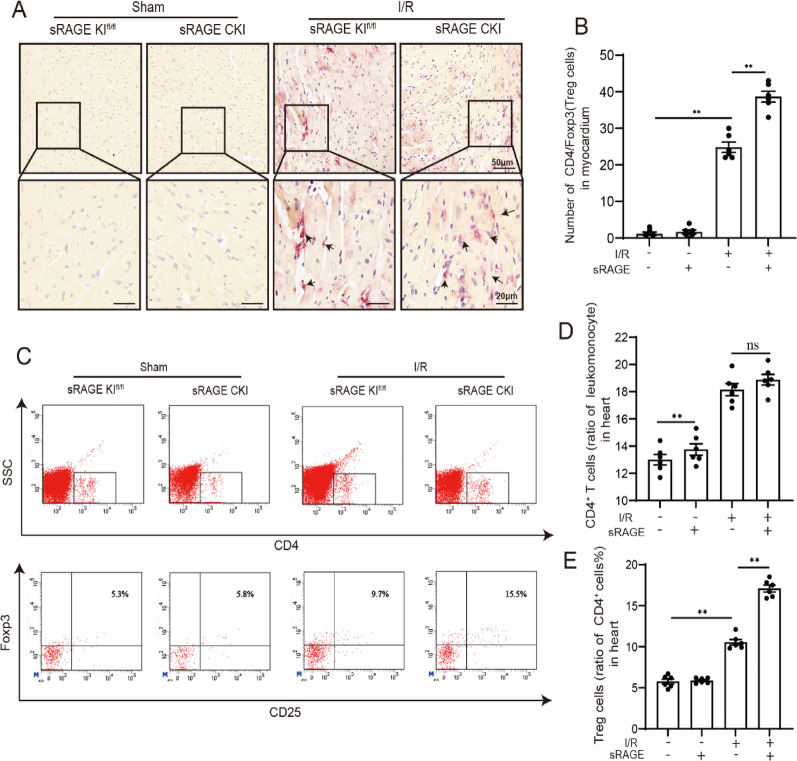
sRAGE increased Tregs in myocardial tissue during I/R.** A** Representative images of CD4 and Foxp3 double staining. CD4 was shown as brown, Foxp3 was shown as red. The scale bars are 50 μm–20 μm. **B** Quantitative data of CD4 and Foxp3 immunohistochemistry double staining in heart tissue, *n* = 6 per group. **C** Representative flow analysis images of Tregs in the mice heart, *n* = 6 per group. **D** Quantitative data of CD4^+^ T cells in heart by flow cytometry. **E** Quantitative data of CD4^+^CD25^+^Foxp3 Tregs in heart by flow cytometry. The data are expressed as the mean ± SEM. **p < 0.01; ns, *p* ≥ 0.05

To explore the potential role of sRAGE increasing the population of Tregs in myocardial tissue during I/R, immunohistochemical double-staining for CD4 and Foxp3 were conducted on the hearts with or without I/R injury in both wild type and sRAEG CKI mice. Western blot analysis confirmed sRAGE overexpression in sRAGE CKI mice (Fig.S1). Compared to the Sham group, the I/R group exhibited a significant increase in CD4⁺Foxp3⁺ double-positive cells (indicative of Tregs), which was further augmented by cardiac sRAGE overexpression (*p* < 0.05, Fig. 1A, B). Similar results for the effects of sRAGE on Tregs were obtained by flow cytometric analysis (*p* < 0.05, Fig. 1C–E). These results suggested that sRAGE attenuated myocardial I/R injury by increasing the number of Tregs in the myocardium.

### Tregs mediated the protective effects of sRAGE on cardiac function

**Fig. 2:**
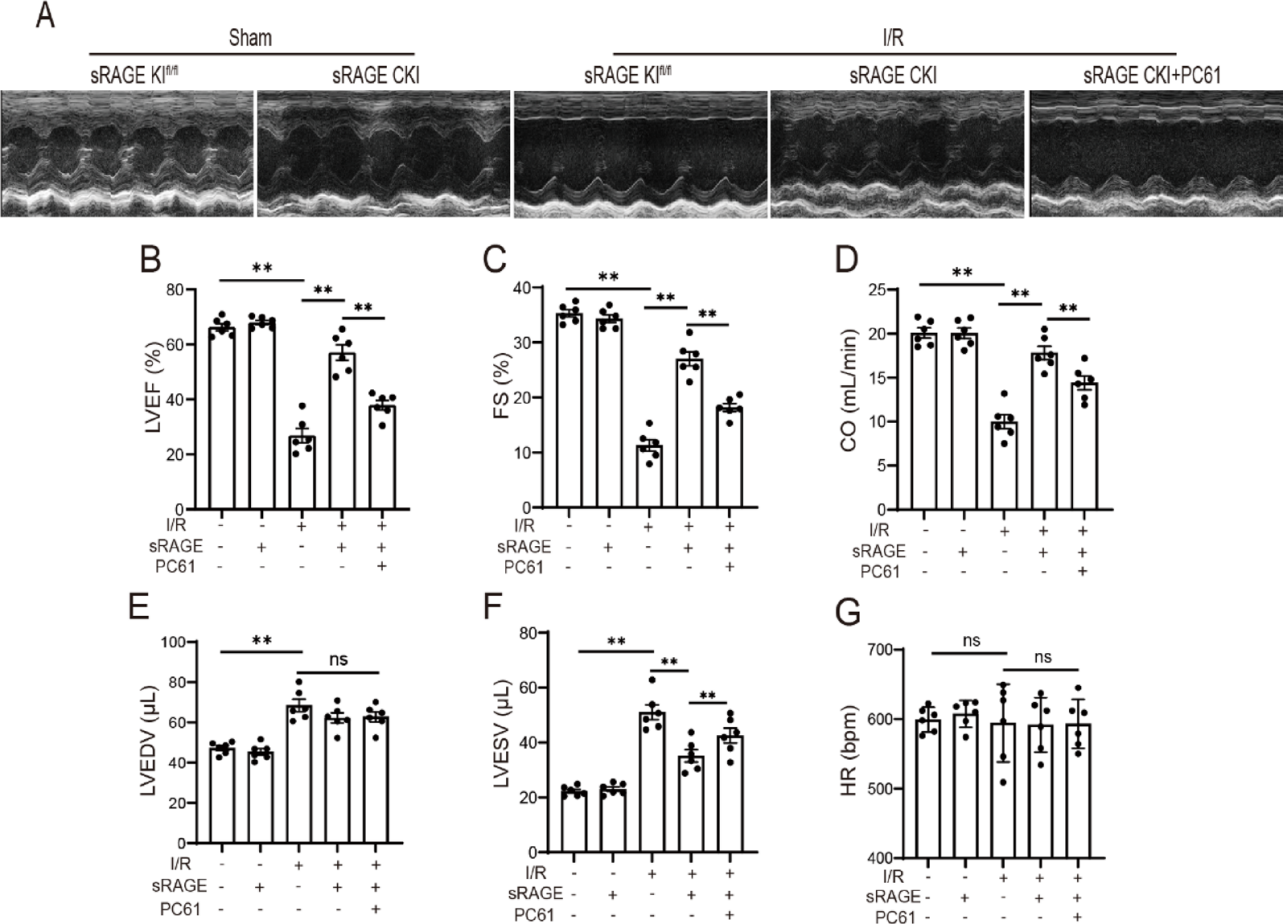
Tregs involved in the protective effects of sRAGE on cardiac function.** A** The representative M-mode echocardiography images of long-axis. **B** Quantitative data of LVEF for each group. **C** Quantitative data of FS for each group. **D** Quantification data of CO for each group. **E** Quantitative data of LVEDV for each group. **F** Quantitative data of LVESV for each group. **G** Quantitative data of HR for each group. The data are expressed as the mean ± SEM. **p < 0.01; ns, *p* ≥ 0.05

To determine whether Tregs are essential for sRAGE-mediated cardio-protection during I/R, cardiac function was evaluated after Tregs depletion using anti-CD25 antibody (PC61) as previously described [24]. The results showed that Cardiomyocyte-specific sRAGE overexpression significantly improved left ventricular anterior wall motion amplitude during I/R, an effect abrogated by PC61 administration (Fig. 2A). PC61 also blunted sRAGE-induced improvements in LVEF, which increased from 26.83 ± 2.63% (post-I/R) to 57.02 ± 2.81% with sRAGE overexpression but decreased to 37.87 ± 1.76% following PC61 treatment (*p* < 0.01, Fig. 2B). Similarly, FS improved from 11.27 ± 1.05% after I/R to 27.00 ± 1.26% following sRAGE overexpression, but was reduced to18.17 ± 0.73% by PC61 (*p* < 0.01, Fig. 2C). CO increased from 9.98 ± 0.79 mL/min to 17.82 ± 0.76 mL/min with sRAGE, but was partially reversed to 14.40 ± 0.71 mL/min by PC61 (*p* < 0.01, Fig. 2D). LVEDV decreased from 68.45 ± 3.06 µL to 62.23 ± 2.51 µL with sRAGE (*p* > 0.05, Fig. 2E), an effect not significantly reversed to 62.77 ± 2.47 µL by PC61 (*p* > 0.05, Fig. 2E). LVESV decreased from 51.05 ± 2.71 µL to 35.17 ± 2.26 µL with sRAGE, but was reversed to 42.52 ± 2.69 µL by PC61 (*p* < 0.01, Fig. 2F). No significant intergroup differences in HR were observed (Fig. 2G). These results suggested that Tregs mediated the protective effects of sRAGE on cardiac dysfunction induced by I/R.

### Tregs involved in the inhibiting effects of sRAGE on I/R-induced cell death

**Fig. 3:**
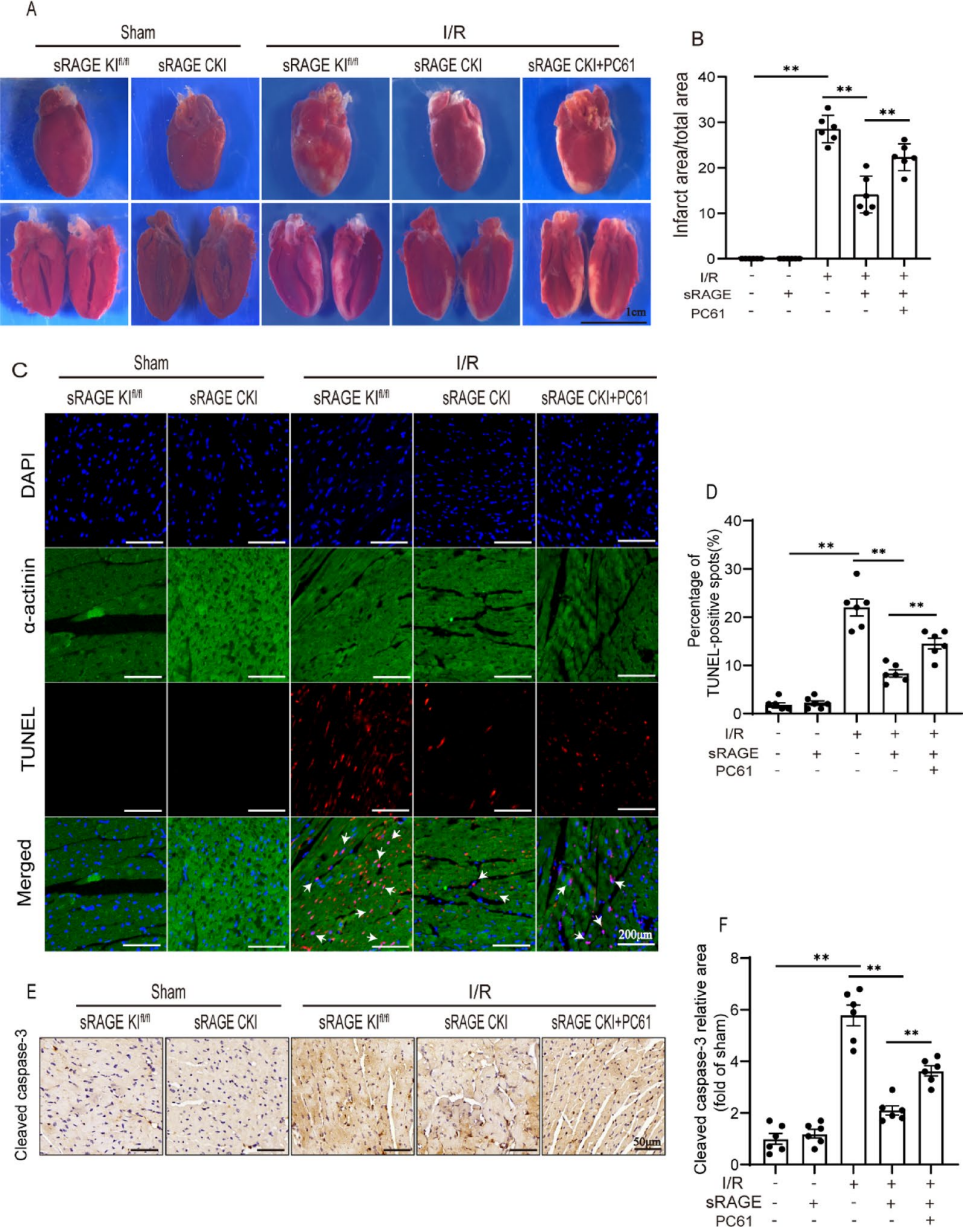
Tregs involved in the inhibiting effects of sRAGE on I/R-induced cell death.** A** The representative images of TTC staining. The scale bars are 1 cm. **B** Quantitative data of the infarct area/total left ventricular area, *n* = 6 per group. **C** Representative images of TUNEL staining. The red spots indicated TUNEL-positive cells. The green fluorescence indicated α-actinin. The blue fluorescence indicated the nuclei. Arrows indicated TUNEL-positive cells. The scale bars are 200 μm. **D** Quantitative data of TUNEL-positive cells, *n* = 6 per group. **E** Representative images of Cleaved caspase-3 immunochemistry staining. The target protein was shown as brown. The scale bars are 50 μm. **F** Quantitative data of Cleaved caspase-3 immunochemistry staining, *n* = 6 per group. The data are expressed as the mean ± SEM. **p < 0.01

To determine whether Tregs contribute to sRAGE-mediated suppression of I/R-induced cell death, infarct area and cell apoptosis were accessed. TTC staining revealed that sRAGE overexpression significantly reduced the infarct area from 28.54 ± 1.24% (post-I/R) to 14.12 ± 1.65% with sRAGE (*p* < 0.01, Fig. 3A, B). This reduction was notably reversed by the administration of PC61, resulting in an infarct area of 22.36 ± 1.21% (*p* < 0.01, Fig. 3A, B). Consistent with this, sRAGE overexpression significantly decreased the number of TUNEL-positive cells and cleaved caspase-3-positive regions during I/R, effects that were reversed by PC61 treatment (*p* < 0.01, Fig. 3C–F). These results indicated that Tregs mediated the inhibitory effect of sRAGE on cell death induced by I/R.

### Tregs involved in the inhibiting effects of sRAGE on myocardial fibrosis

**Fig. 4:**
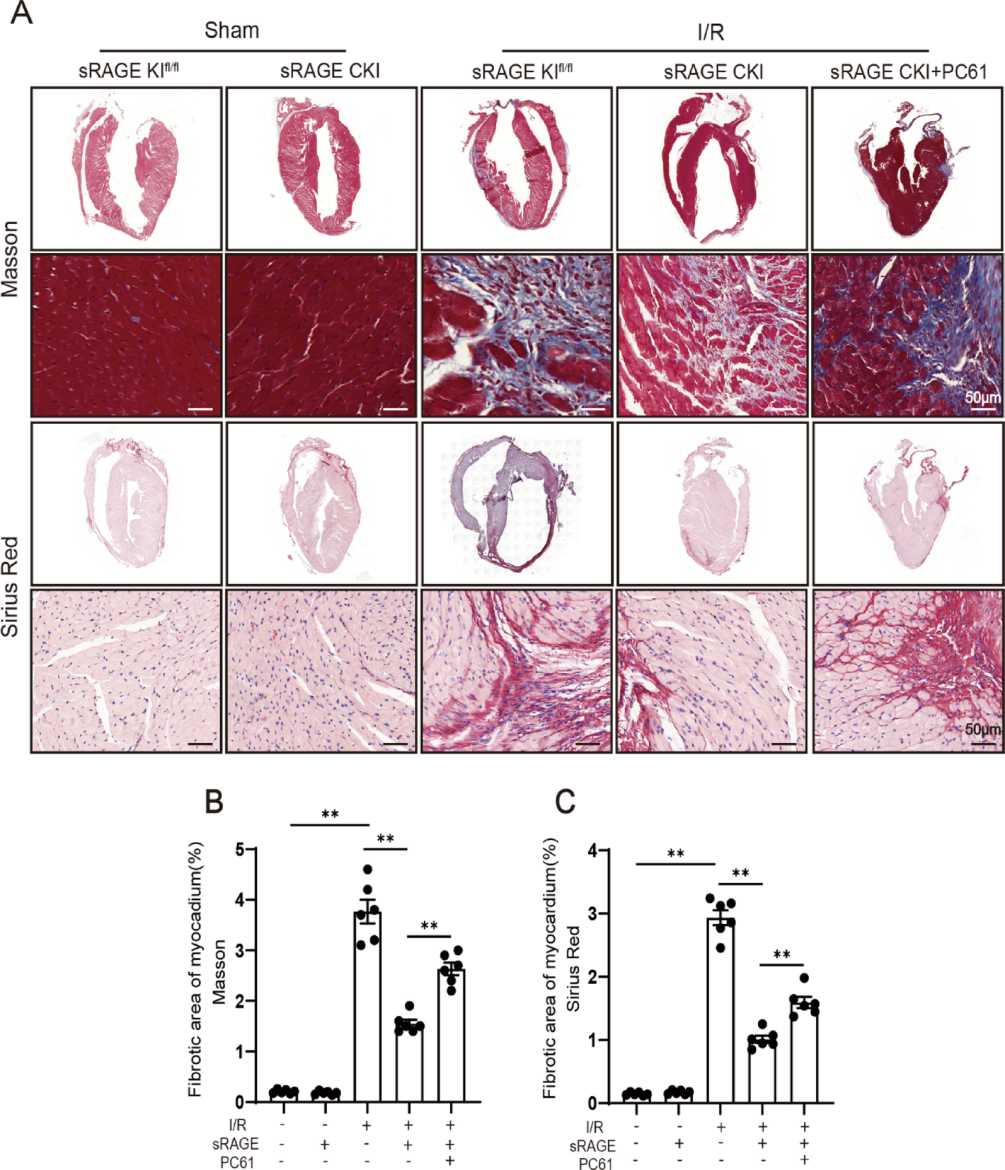
Tregs involved in the inhibiting effects of sRAGE on myocardial fibrosis.** A** Representative images of Masson and Sirius red staining of cardiac tissue. Scale bars are 50 μm. **B** Quantitative data of Masson staining. **C** Quantitative data of Sirius red staining, *n* = 6 per group. The data are expressed as the mean ± SEM. **p < 0.01

To further elucidate the role of Tregs in the sRAGE-mediated inhibition of I/R-induced myocardial fibrosis, Masson staining and Sirius red staining were performed. I/R injury significantly increased myocardial fibrosis, which was potently reduced by sRAGE overexpression (*p* < 0.01, Fig. 4). Conversely, Tregs depletion with PC61 completely reversed the antifibrotic effects of sRAGE (*p* < 0.01, Fig. 4). These results suggested that Tregs mediated the inhibitory effects of sRAGE on I/R-induced myocardial fibrosis.

### Tregs involved in the inhibiting effects of sRAGE on inflammatory response

**Fig. 5:**
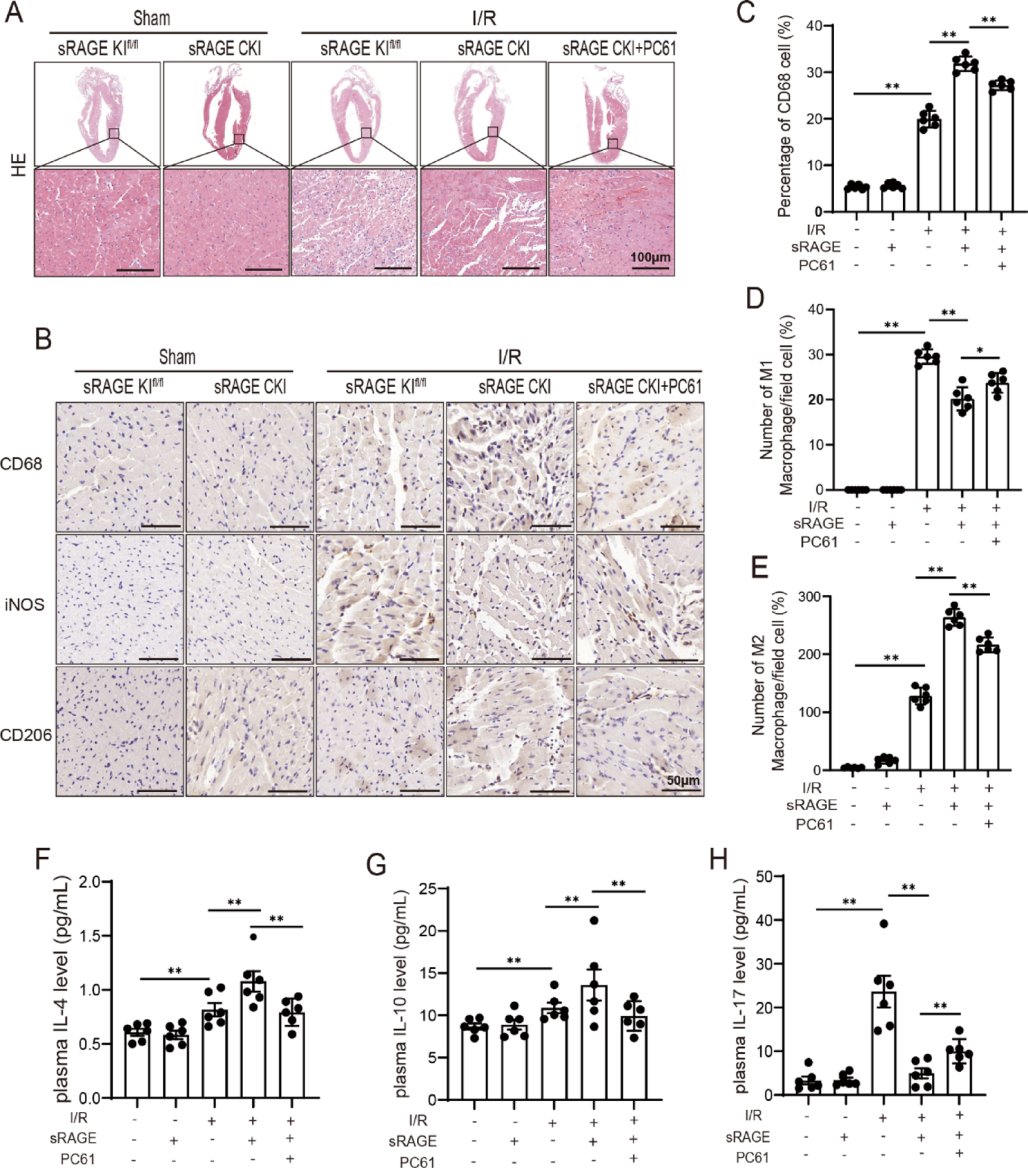
Tregs involved in the inhibiting effects of sRAGE on inflammatory response.** A** Representative images of HE staining of heart sections. The scale bars are 100 μm. **B** Representative images of CD68, iNOS and CD206 staining. The scale bars are 50 μm. **C** Quantitative data of CD68 (macrophage marker) positive cells. **D** Quantitative data of iNOS (M1 macrophage marker) positive cells. **E** Quantitative data of CD206 (M2 macrophage marker) positive cells. **F** Quantitative data of plasma IL-4 level, *n* = 6 per group. **G** Quantitative data of plasma IL-10 level, *n* = 6 per group. **H** Quantitative data of plasma IL-17 level, *n* = 6 per group. The data are expressed as the mean ± SEM, *p < 0.05, **p < 0.01

To determine whether Tregs are involved in sRAGE-mediated inhibition of I/R-induced inflammation, we evaluated macrophage infiltration and inflammatory cytokine levels. HE staining revealed that sRAGE overexpression reduced I/R-induced inflammatory cell infiltration in cardiac tissue, an effect was reversed by PC61 treatment (Fig. 5A). Immunohistochemical analysis showed a significant increase in the numbers of CD68-positive (macrophage marker) cells following sRAGE overexpression during I/R, which were reversed by PC61 (*p* < 0.01, Fig. 5B, C). The numbers of iNOS-positive (M1 macrophage marker) cells significantly decreased following sRAGE overexpression during I/R (*p* < 0.01, Fig. 5B, D), which were reversed by PC61 administration (*p* < 0.05, Fig. 5B, D). Conversely, the numbers of CD206-positive (M2 macrophage marker) cells significantly increased with sRAGE overexpression during I/R, but decreased upon PC61 treatment (*p* < 0.01, Fig. 5B, E). ELISA showed that sRAGE overexpression upregulated anti-inflammatory cytokines IL-4 and IL-10 during I/R, which were reversed by PC61 administration (*p* < 0.01, Fig. 5F, G). In contrast, the pro-inflammatory cytokine IL-17 was reduced after sRAGE overexpression during I/R, but increased with PC61 administration (*p* < 0.01, Fig. 5H). These results suggested that Tregs mediated the inhibitory effects of sRAGE on inflammatory response induced by I/R.

### sRAGE increased the expression of PD-L1 in cardiomyocytes

**Fig. 6:**
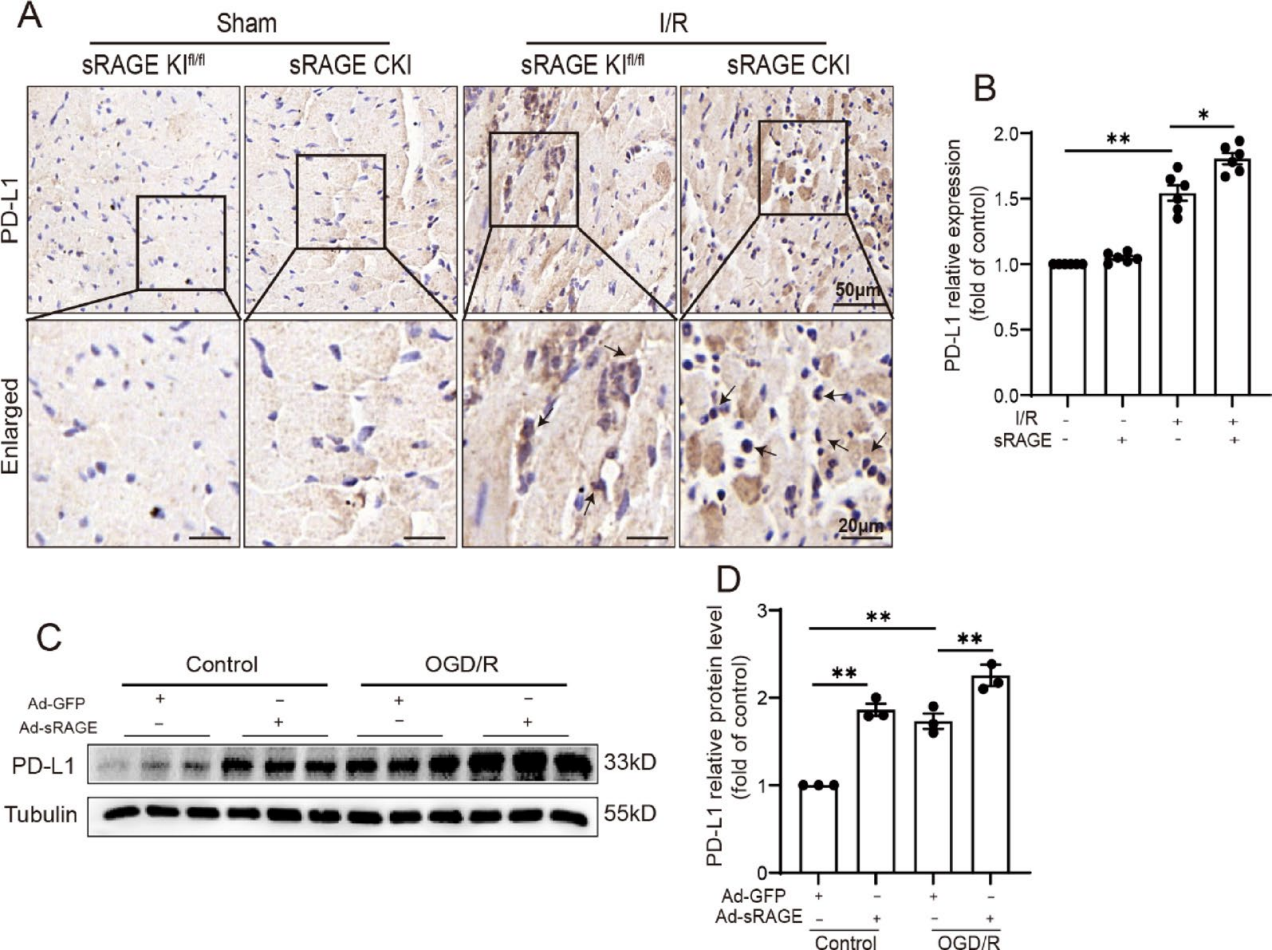
sRAGE increased the expression of PD-L1 in cardiomyocytes.** A** Representative images of PD-L1 immunohistochemical staining. The scale bars are 50 μm–20 μm. **B** Quantitative data of PD-L1 immunohistochemical staining, *n* = 6 per group. **C** Representative images of Western blotting for PD-L1 in cardiomyocytes. **D** Quantitative data of the Western blotting results for PD-L1, *n* = 3 per group. The data are expressed as the mean ± SEM, *p < 0.05, **p < 0.01

Given that PD-L1 has been reported to facilitate the differentiation of CD4^+^ T cells into Tregs, the impact of sRAGE on PD-L1 expression in cardiomyocytes during I/R was assessed. Immunohistochemistry showed significantly increased PD-L1-positive areas in the I/R group, further enhanced by sRAGE overexpression (*p* < 0.05, Fig. 6A, B). Consistent results were obtained from the Western blot analyses of cardiomyocytes subjected to OGD/R in vitro (*p* < 0.01, Fig. 6C, D). These results suggested that sRAGE could increase the expression of PD-L1 in cardiomyocytes during I/R both in vivo and in vitro.

### Inhibition of PD-L1 decreased the effect of sRAGE on Tregs

**Fig. 7:**
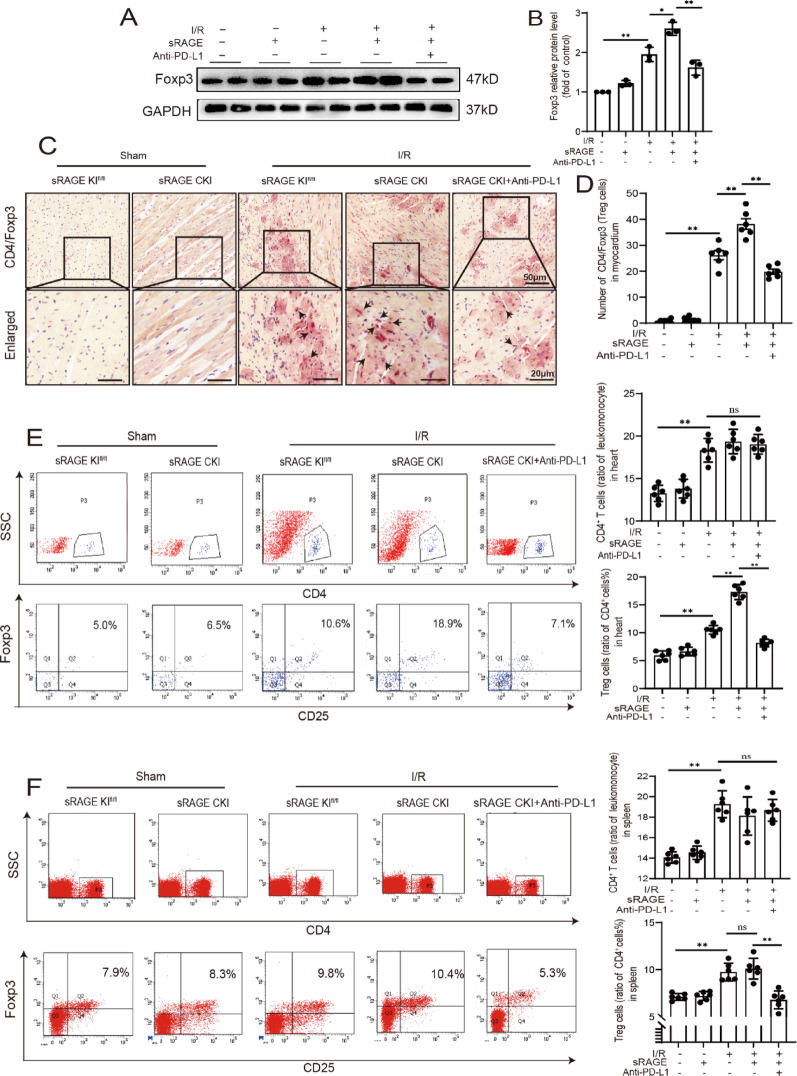
Inhibited PD-L1 decreases the effect of sRAGE on Tregs.** A** Representative images of Western blot for Foxp3. **B** Quantitative data of the Western blotting results for Foxp3, *n* = 3 per group. **C** Representative images of CD4 and Foxp3 double staining. CD4 was shown as brown, Foxp3 was shown as red. The scale bars are 50 μm–20 μm. **D** Quantitative data of CD4 and Foxp3 immunohistochemistry double staining, *n* = 6 per group. **E** The proportions of CD4^+^T cells and Tregs in heart tissue were detected by flow cytometry. *n* = 6 per group. **F** The proportions of CD4^+^T cells and Tregs in the spleen tissue by flow cytometry, *n* = 6 per group. The data are expressed as the mean ± SEM, *p < 0.05; **p < 0.01; ns, *p* ≥ 0.05

To further assess the influence of PD-L1 on Tregs in cardiomyocytes after sRAGE treatment, PD-L1/PD-1 interaction was blocked using a PD-L1 antibody. Both Western blot and immunohistochemical analyses revealed that sRAGE overexpression significantly increased Foxp3 protein (a specific biomarker for Tregs) expression in the myocardium during I/R, an effect reversed by PD-L1 antibody treatment. (*p* < 0.05, Fig. 7A–D). Consistent results were obtained from the flow cytometric analysis, PD-L1 antibody significantly altered the proportion of Tregs in cardiac tissue. Specifically, the percentage of these cells increased from 10.78 ± 0.23% following I/R to 17.45 ± 0.44% with overexpressing sRAGE (*p* < 0.01, Fig. 7E). This increase was subsequently reduced to 7.73 ± 0.20% upon treatment with PD-L1 antibody (*p* < 0.01, Fig. 7E). In spleen tissue, while sRAGE had no significant effect on Tregs proportion (9.68 ± 0.23% vs. 10.10 ± 0.21%, *p* > 0.05, Fig. 7F), PD-L1 antibody significantly decreased it to 6.78 ± 0.39% (*p* < 0.01, Fig. 7F). These results suggested that PD-L1 played a role in the enhancement effects of sRAGE on Tregs in the heart during I/R.

### sRAGE promoted the differentiation of CD4^+^ T cells into Tregs during I/R

**Fig. 8:**
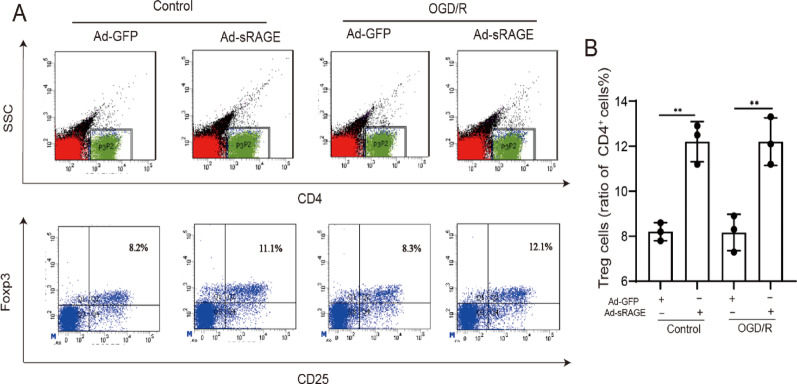
sRAGE promoted the differentiation of CD4^+^ T cells into Tregs during I/R.** A** Representative flow analysis images of Tregs. **B** Quantitative data of Tregs by flow cytometry, *n* = 3 per group. The data are expressed as the mean ± SEM. **p < 0.01

To clarify how sRAGE enhanced Tregs numbers during I/R, primary naïve CD4^+^ T cells were isolated from mouse spleens and co-cultured with neonatal rat cardiomyocytes. Co-culture with sRAGE-overexpressing cardiomyocytes significantly increased Tregs differentiation from CD4⁺ T cells, both under normoxic conditions and after OGD/R. Specifically, the numbers of Tregs were increased from 8.20 ± 0.23% to 12.20 ± 0.51% under normoxia (*p* < 0.01, Fig. 8) and from 8.17 ± 0.47% to 12.20 ± 0.61% after OGD/R (*p* < 0.01, Fig. 8). These results suggested that sRAGE might promote the differentiation of CD4^+^ T cells into Tregs during I/R.

### Inhibition of JAK2/STAT3 pathway attenuated the effect of sRAGE on the up-regulation of PD-L1

**Fig. 9:**
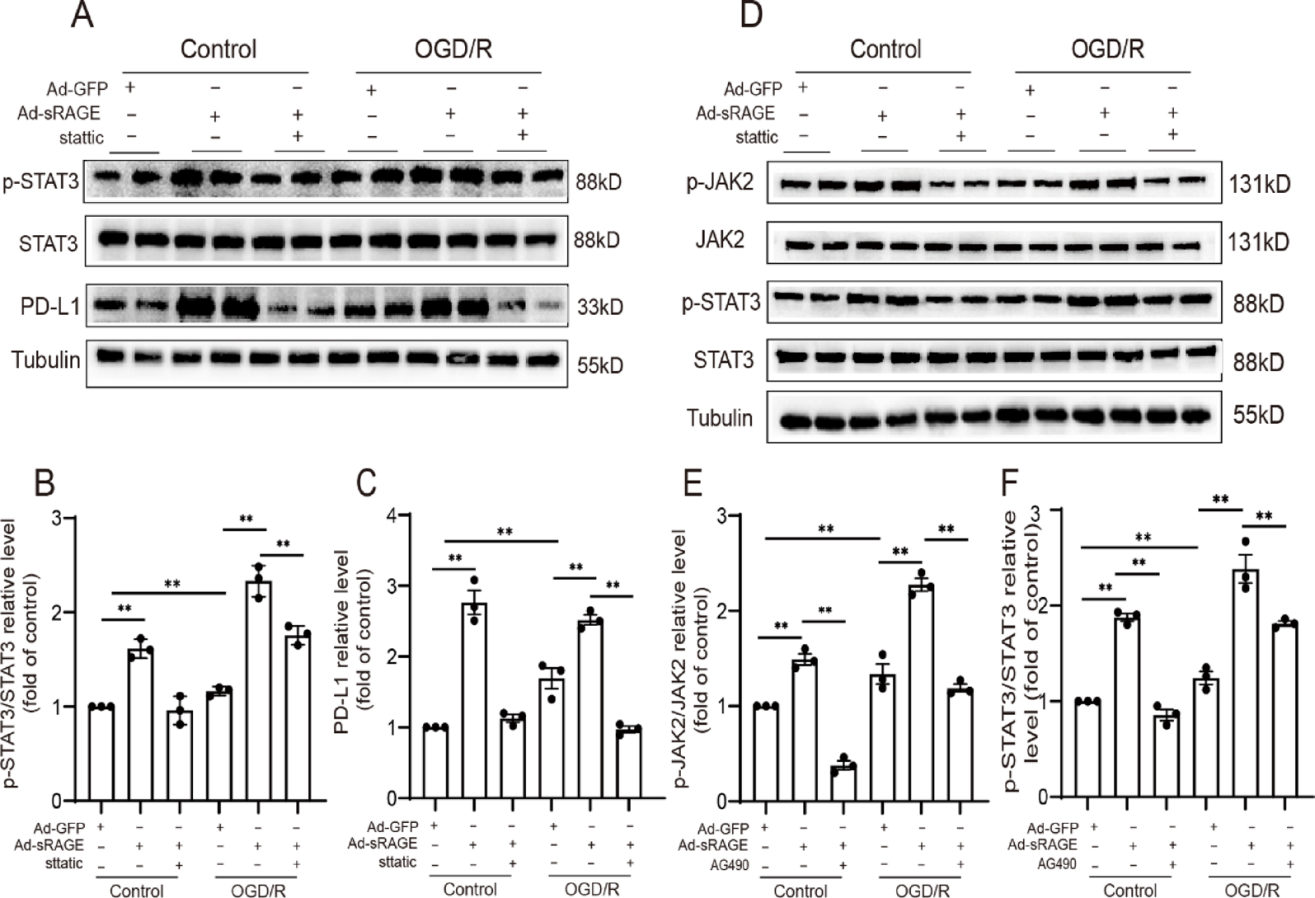
sRAGE regulated the expression of PD-L1 through JAK2-STAT3 pathway.** A** Representative images of Western blotting for p-STAT3, STAT3, PD-L1 of each group. **B** Quantitative data of the Western blotting results for p-STAT3/STAT3. **C** Quantitative data of the Western blotting results for PD-L1. **D** Representative images of Western blotting for p-JAK2, JAK2, p-STAT3, STAT3 of each group. **E** Quantitative analysis of the Western blotting results for p-JAK2/JAK2. **F** Quantitative analysis of the Western blotting results for p-STAT3/STAT3. *n* = 3 per group. The data are expressed as the mean ± SEM. **p < 0.01

Given that the JAK/STAT3 pathway has been implicated in the transcription regulation of PD-L1 [20], JAK2 inhibitor AG490 and STAT3 inhibitor stattic were employed to elucidate the role of JAK2/STAT3 in modulating PD-L1 expression in sRAGE-treated cardiomyocytes. The results showed that stattic effectively abolished sRAGE-induced PD-L1 upregulation in OGD/R-treated cardiomyocytes (*p* < 0.01, Fig. 9A–C). Similarly, AG490 significantly attenuated the enhancement of JAK2 and STAT3 activity prompted by sRAGE (*p* < 0.01, Fig. 9D–F). Together, these results indicated that sRAGE regulated PD-L1 expression via the JAK2/STAT3 signaling pathway.

## Discussion

The present study demonstrated that sRAGE exerted protective effects against I/R-induced myocardial cell death, fibrosis, and inflammatory responses by increasing the numbers of Tregs in cardiac tissue. This protective mechanism was mediated through the promotion of CD4^+^ T cell differentiation into Tregs, a process driven by the upregulation of PD-L1 expression in cardiomyocytes following the activation of the JAK2/STAT3 signaling pathway.

Initially, the present study identified sRAGE as an endogenous cardio-protective factor in myocardial I/R injury, increasing the numbers of Tregs in cardiac tissue. The hypothesis was substantiated by the results that overexpression of sRAGE in cardiomyocytes led to a significant increase in CD4 and Foxp3 double-positive cells, identified as Tregs, within myocardial tissue during I/R (Fig. 1A, B). These results were further verified by flow cytometric analysis (Fig. 1C–E). Hence, it’s suggested that sRAGE could increase the number of Tregs, potentially serving as a mechanism for mitigating myocardial I/R injury.

Tregs have been documented to accumulate in damaged myocardial tissue, where they secrete anti-inflammatory cytokines, such as IL-4 and IL-10, and promote the polarization of M1 macrophages to M2 phenotypes, thereby attenuating inflammatory responses and cardiac remodeling [6, 7]. In this study, Tregs depletion using anti-CD25 antibody (PC61) significantly abrogated sRAGE-mediated improvements in cardiac function, reduction in myocardial fibrosis, inhibition of cell death, and suppression of inflammation (Figs. 2, 3, 4 and 5). These results suggested that Tregs were integral to mediating the protective effects of sRAGE in myocardial I/R injury.

A previous study indicated that the conversion of conventional CD4^+^ T cells constitutes a source of Tregs in the heart during myocardial I/R injury [7], which was associated with the PD-1 expressed on immunocytes and PD-L1 expressed on non-hematopoietic cells, such as cardiomyocytes [17, 18, 25]. The present study showed that overexpression of sRAGE increased PD-L1 levels in cardiomyocytes both in vivo and in vitro (Fig. 6). Additionally, blocking the PD-L1/PD-1 interaction with a neutralizing antibody reversed the effect of sRAGE on increasing the number of Tregs in heart tissue (Fig. 7). Therefore, sRAGE may directly regulate PD-L1 expression in cardiomyocytes and promote the differentiation of Tregs during myocardial I/R injury.

To further confirm that sRAGE drives CD4⁺ T cell polarization toward Tregs, primary naïve CD4^+^ T cells were isolated and co-cultured with cardiomyocytes overexpressing sRAGE. The results showed that sRAGE increased the numbers of Tregs, which were obviously differentiated from CD4^+^ T cells with or without OGD/R treatment (Fig. 8). Taken together, these findings provide evidence that sRAGE may promote the polarization of CD4^+^ T cells towards Tregs by directly modulating PD-L1 expression in cardiomyocytes during myocardial I/R.

Multiple studies have demonstrated that PD-L1 is transcriptionally regulated by STAT3, a key factor in mitigating excessive inflammation and cell apoptosis during myocardial I/R injury [20, 26]. The classical mechanism for activating STAT3 involves its phosphorylation by JAK2, followed by its translocation to the nucleus, where it regulates the transcription of downstream genes [27]. In the present study, the inhibition of either JAK2 or STAT3 significantly attenuated the effect of sRAGE on the upregulation of STAT3 or PD-L1 expression in cardiomyocytes (Fig. 9). Therefore, it can be concluded that sRAGE-regulated PD-L1 expression through JAK2/STAT3 signaling pathway.

## Conclusion

In summary, this study elucidated a novel mechanism by which sRAGE alleviated myocardial I/R injury. Specifically, sRAGE promoted the differentiation of CD4^+^ T cells into Tregs through the upregulation of PD-L1 expression in cardiomyocytes via a JAK2/STAT3-dependent pathway. This research established a connection between endogenous small molecule substances and Tregs-mediated immune regulation, providing new insights for the therapeutic perspectives for the management of myocardial I/R injury.

## Supplementary Information

Below is the link to the electronic supplementary material.

Supplementary Material 1

Supplementary Material 2

## Data Availability

The datasets supporting the conclusions of this article are included within the article and its additional files.

## Abbreviations

AMI: Acute myocardial infarction.
CO: Cardiac output
FS: Shortening fraction
HR: Heart rate
I/R: Ischemia/reperfusion
JAK2: Janus kinase 2
LVEF: Left ventricular ejection fraction
LVEDV: Left ventricular end-diastolic volume
LVESV: Left ventricular end-systolic volume
LSD: Least significant difference
OGD/R: Glucose deprivation/reoxygenation
PD-1: Programmed death-1
PD-L1: Programmed cell death ligand 1
PBS: Phosphate-buffered saline
sRAGE: Soluble receptor for advanced glycation end-products
sRAGE-CKI: Cardiomyocyte-specifc sRAGE knock-in
STAT: Signal transducers and activators of transcription
Tregs: Regulatory T cells
TTC: Triphenyltetrazolium chloride
