# Supplementary material for: sRAGE inhibits myocardial ischemia/reperfusion injuries via regulating Treg cells
Source: Cell Biosci. 2025 Oct 22;15:144. doi: 10.1186/s13578-025-01482-y (PMC12542090; doi:10.1186/s13578-025-01482-y)
Supplement: Supplementary file 1 — Supplementary Material 1 [file 13578_2025_1482_MOESM1_ESM.docx]

**Supplementary Information**

**sRAGE Inhibits Myocardial Ischemia/Reperfusion Injuries via Regulating Treg cells**

Content:

1. Supplementary Tables

2. Supplementary Figures and Figure Legends

**Supplementary Table 1. Sequences of the mice genotyping and real-time PCR.**

| Mice genotyping | |
| --- | --- |
| sRAGE-F | AGATTCTGCCTCTGAACTCACG |
| sRAGE-R | GGGTCACCATTAGCTCCGAC |
| WT-F | CACTTGCTCTCCCAAAGTCGCTC |
| WT-R | ATACTCCGAGGCGGATCACAA |

**Supplementary Table 2. List of antibodies with their sources and experimental conditions.**

| Marker | Species | Application | Manufacturer | Catalog No. | Dilution |
| --- | --- | --- | --- | --- | --- |
| anti-PD-L1 | Rabbit | IHC | Thermo | Thermo | 1:200 |
|  |  | WB | Thermo | sl90880 | 1:1000 |
| anti-CD68 | Rabbit | IHC | Abcam | Ab125212 | 1:200 |
| anti-iNOS | Rabbit | IHC | Abcam | Ab15323 | 1:200 |
| anti-CD206 | Rabbit | IHC | Abcam | Ab64693 | 1:200 |
| anti-cleaved caspase-3 | Rabbit | IHC | CST | 9664 | 1:200 |
| anti-pSTAT3 | Rabbit | WB | CST | 9131 | 1:1000 |
| anti-STAT3 | Rabbit | WB | CST | 4904 | 1:1000 |
| anti-pJAK2 | Rabbit | WB | CST | 238791 | 1:1000 |
| anti-JAK2 | Rabbit | WB | CST | 40648 | 1:1000 |
| anti-Tubulin | Rabbit | WB | CST | 38789 | 1:1000 |
| anti-GAPDH | Rabbit | WB | CST | 30098 | 1:1000 |

**Supplemental Figure 1**

**
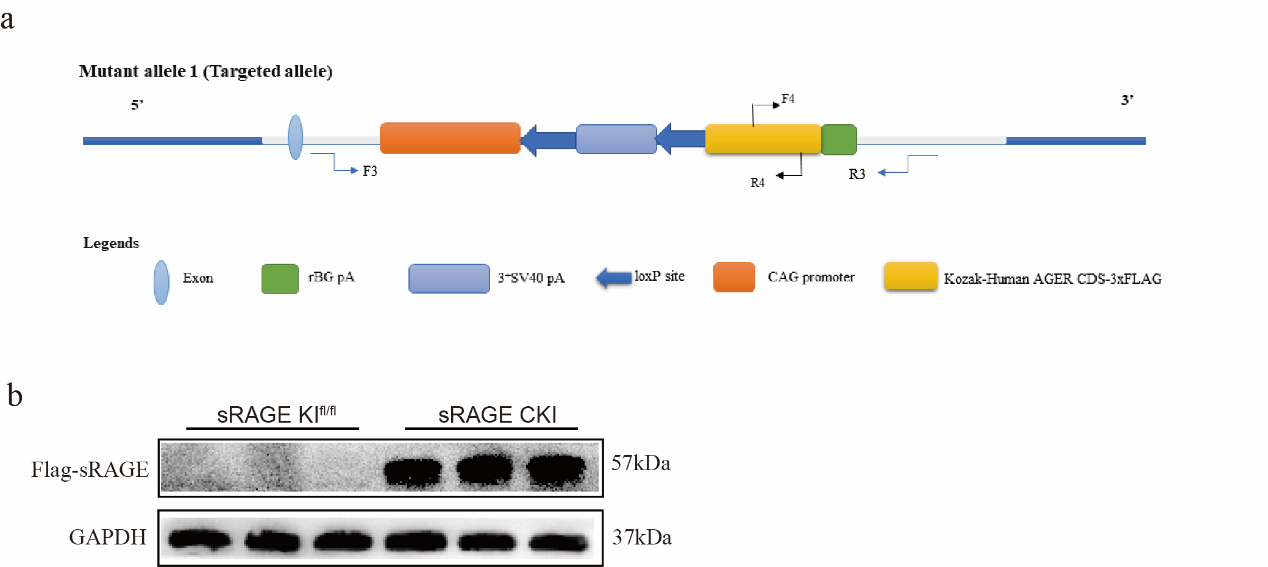
**

**Fig. S1** sRAGE CKI mice with high expression of sRAGE protein. **a** Diagram of the humanized sRAGE allele knock-in mice constructs. **b** Representative images of Western blotting for sRAGE in mice.
